# After the Crisis: Explaining stories of professional identity growth from collective action

**DOI:** 10.1177/01708406241295495

**Published:** 2024-11-11

**Authors:** Derin Kent, M. Tina Dacin

**Affiliations:** University of Warwick, UK; Queen’s University, Canada

**Keywords:** collective action, crisis, expertise, professional identity, self-narratives

## Abstract

Emergent forms of collaboration are central to societies’ response to crises like natural disaster, refugee migration and pandemics. Even though individuals’ participation in such collective action may be short-lived, recent studies propose it can inspire enduring professional role change as people return to their everyday work post-crisis. Yet, previous research does not focus on the divergent stories participants tell about the crisis years afterward and what it meant for them professionally. Through a grounded study of healthcare workers involved in the 2003 Toronto SARS outbreak, we examine varied narratives of professional identity growth following collective action in crisis. Years after SARS, participants told diverse stories about the crisis as an event that suspended, affirmed or even expanded their professional identities. Participants with narratives of identity suspension saw SARS as an event lacking professional relevance. Narratives of identity affirmation and expansion, however, emphasized growth and inspiration for participants’ professional roles post-crisis. We theorize how interactions within collective responses can foster growth narratives, when they enhance meanings central to participants’ professional identities and by affording follow-on interactions that translate these meanings into role change. We contribute new insight on how collective action in crisis can lead to professional role change post-crisis, how fragmented perspectives affect capacity for collective action in intermittent crises, and the role of follow-on interactions in professionals’ narrative identity work.

## Introduction

Emergent forms of collaboration are central in societies’ response to intermittent crises such as natural disaster, refugee migration and pandemics which can catch organizations, communities and institutions ill prepared at their onset (Kornberger, 2022; Majchrzak, Jarvenpaa, & Hollingshead, 2007). As the literature on collective action in crisis reveals, distributed actors improvise responses when faced with a ‘massive, visible, shared problem’, with spontaneous collaboration among professionals within and across organizations playing an especially important role (Beck & Plowman, 2014, p. 1238; Kornberger, Leixnering, & Meyer, 2019). While these emergent collaborations may be short-lived, new research is beginning to show that collective action can inspire enduring professional growth for the participants involved as they return to their everyday work in organizations post-crisis (Compagni, Cappellaro, & Nigam, 2024). This is a valuable insight because it means that those who respond to a societal crisis may come out of it more resilient in their professional roles and in their capacity to engage in collective action in future crises.

How participating in collective action in crisis may lead to professional growth among organizational participants post-crisis presents a puzzle. Recent studies theorize that collective action can lead individuals to change how they enact their professional roles post-crisis by exposing them to new practices and perspectives about their work role that come to be associated with positive meanings or emotions (Compagni et al., 2024; Jiang, 2021; Sahay & Dwyer, 2021). But while joining in the response to an event like a pandemic or war can be a profound personal experience (Wright, Meyer, Reay, & Staggs, 2021) it does not necessarily lead to the institutional changes usually associated with professional role change in organizations and institutions (Chreim, Williams, & Hinings, 2007; Goodrick & Reay, 2010; Wright, Nyberg, & Grant, 2012). This raises the question of how professionals come to integrate practices and perspectives gained during collective action into their professional roles as they resettle into everyday work.

In this paper, we study professional growth among participants of collective action in crisis. We examine the stories they tell about the crisis and what it means for their professional identity. Specifically, we ask, *How do participants develop narratives of professional identity growth from a collective response to a societal crisis?* We take a narrative approach because as professionals carry on with their careers in the months and years afterward, their attempts to make sense are likely to generate personalized narratives about the crisis, ones which integrate disparate experiences into coherent themes such as survival, recovery or growth (Ibarra & Barbulescu, 2010; Vough & Caza, 2017). We are interested in narratives of identity growth because these are likely to encourage individuals to integrate practices and perspectives from the crisis into their professional roles in ways that will contribute to their professional field and its capacity to handle future crises. Conversely, narratives of helplessness and disillusionment lead towards disengagement (Gabriel, Gray, & Goregaokar, 2010), something likely to weaken collective capacity in future crises by depriving responses of experienced participants.

We conducted a theory-building study of collective responses within and across Toronto hospitals to the 2003 SARS outbreak. Years afterward, staff made sense of the crisis in diverse ways as an event that suspended, affirmed or expanded their professional identities. We theorize how interactions within and after collective action can foster growth narratives that inspire different forms of post-crisis professional role change. Our study contributes to the literature on collective action by providing insight on professional role change that arises from participants’ involvement with a crisis, showing how identity narratives help explain both the endurance and varieties of professional growth that arise post-crisis (Compagni et al., 2024; Jiang, 2021). By emphasizing the divergent stories professionals tell about growth, we extend scholars’ understanding of the fragmentation of perspectives in crisis response and explain how this can affect the capacity for collective action in future crises (Wolbers, Boersma, & Groenewegen, 2018). We also contribute to the literature on narrative identity work in careers (Vough & Caza, 2017) by highlighting how people get entwined in different follow-on collegial interactions after collective disruptions that direct their narrative work towards professional paths that encourage either a specialist or generalist identity. Overall, even though crises are collective events, our view is that professionals engage with them in dissimilar ways within the collective response and come out with divergent narratives about professional growth.

## Professional Identity Growth from Collective Action in Crisis

### Professional role change from collective action in crisis

Recent events such as pandemics, refugee migration and wars across the globe have encouraged scholars to study the experiences of professionals who participate in collective responses to crisis, including healthcare workers containing Covid-19 and Ebola (Wright et al., 2021), staff collaborating within and across agencies to manage mass migration of refugees (Kornberger et al., 2019), and engineers working across organizational boundaries to contain a spaceflight disaster (Beck & Plowman, 2014). In such responses, professionals participate in a joint effort to resolve a public problem that falls within their jurisdiction but for which existing solutions are poorly suited to handle (Abbott, 1988; Kornberger et al., 2019). Because of the scale and novelty of such problems, the response typically requires professionals to make temporary but drastic adaptions to their roles, ranging from individual changes to their routines to collaborating in temporary, interorganizational structures (Beck & Plowman, 2014; Wright et al., 2021).

Scholars’ interest in how these experiences shape the work of professionals once the crisis – or at least their involvement with it – has ended represents an important development in the literature on collective action in crisis. The traditional focus has been on investigating the modes by which collective action arises among distributed actors responding to a crisis (Kornberger, 2022). As urgent and shared problems, societal crises enable forms of collaboration among actors that would not otherwise occur in daily organizational life (Kornberger et al., 2019). Yet, by moving to the level of individual participants’ experiences, new research proposes that participation in these temporary collaborations may give individuals enduring insights into their professional roles once the crisis has settled (Compagni et al., 2024). This is a valuable insight, not least because if the lessons and collaborations developed in one crisis are appreciated and cultivated by the people who responded to it, their organizations and societies will be better prepared to handle the next crisis (Dwyer & Hardy, 2016). Yet, much remains to be understood about how collective action in crisis informs professional role change among participants post-crisis.

A key puzzle is how participants integrate the practices and perspectives they have gained in acute crises into their everyday professional roles. While there is an established literature on how institutional change (Chreim et al., 2007; Goodrick & Reay, 2010) or social movements like those associated with climate change can reshape professional identities (Lefsrud & Meyer, 2012; Wright et al., 2012), collective action in acute crises tends to leave participants returning to their daily work without a shared template for change. In the absence of institutional-level change, recent research proposes that participating in a collective response may nevertheless inspire post-crisis growth by disrupting core meanings associated with professionals’ roles. For instance, Compagni et al. (2024, p. 829) conceptualize healthcare workers’ collective action during Covid-19 as a ‘knowledge disruption’, showing that participants who find their expert status undermined may emphasize instead the value of collegial and humanistic work. Likewise, Jiang (2021, p. 819) shows how refugee resettlement workers adopted a ‘situational purpose’, emphasizing the value of helping a large number of refugees when a surge made it difficult to sustain quality interactions. The idea is that participants reconceptualize their work to uphold positive meanings such as moral worth and competence as they adapt to new demands (Sahay & Dwyer, 2021). Positive associations with these new practices are then theorized to encourage professionals to continue with them after the crisis, strengthening for instance service-oriented (Compagni et al., 2024) or values custodianship practices among physicians post-pandemic (Wright et al., 2021).

Yet, the short observation periods of existing studies make it difficult to see whether participants’ immediate appreciation for new practices or perspectives on their professional role endures in the years ahead as they and their institutions settle back into a new state of normal. As Compagni et al. (2024, p. 857) note, it remains unclear how ‘long lasting [professionals’] responses might actually be and their long-term repercussion on the profession. . . for instance whether the [new practices] [persist] over time’. This is a significant issue because the meanings professionals give to a crisis retrospectively can diverge from their initial impressions and be shaped by interactions long after (Weick, 1995). Further, studies do not yet address the contrasting lessons professionals can draw from the crisis, a strong possibility since crisis responses can generate fragmented perspectives among participants taking on different roles (Wolbers et al., 2018). Thus, participants may reach different conclusions about whether the events disrupted core meanings associated with their profession (Rapp, Hughley, & Kreiner, 2024). We propose that understanding the insights professionals draw from collective responses requires us to examine the meaning making that occurs after the crisis. To do this, we draw on the concept of professional identity narratives.

### Professional identity narratives, disruption and growth

Professional identity refers to ‘an individual’s self-definition as a member of a profession’ (Chreim et al., 2007, p. 1515). As an aspect of identity, it consists of meanings that define oneself constructed from personal attributes and membership in social groups. We focus on growth in individuals’ professional role identities, those identities that inform how individuals carry out their professional role: how they define themselves in that role and what aspects of their profession they value and uphold (Ashforth, 2001, p. 6). Professional role identities are shaped by both institutional elements such as professional associations that specify how one can enact their professional role as well as individuals’ identity work aimed at claiming, revising and altering their identities (Chreim et al., 2007).

Self-narratives represent a valuable lens to explore professional role identity growth following collective action in crisis. They are stories ‘that [make] a point about the narrator’ and, in doing so, help individuals construct and express who they are professionally (Ibarra & Barbulescu, 2010, p. 135). Narratives create meaning by putting one’s career in temporal sequence, creating a logical and plausible connection between events to express coherent themes such as discovery, growth or stagnation. It is through narratives that people give meaning to change in their career and what significance it has in the present (Vough & Caza, 2017). Through such stories people understand, craft and enact their professional role identity, including the beliefs, values and interaction styles that underlie it (Ashforth, 2001; Croft, Currie, & Lockett, 2015). The literature on professional identity shows that professionals revise their self-narratives to accommodate changes ranging from career transitions (Pratt, Rockmann, & Kaufmann, 2006) to institutional and profession-wide changes that direct members to collectively adapt their roles (Goodrick & Reay, 2010; Lefsrud & Meyer, 2012; Wright et al., 2012).

Events that disrupt people’s professional self-narratives can result in change and even growth in their professional identity (Obodaru, 2017; Shepherd & Williams, 2018). Events such as job loss or radical career transitions break the logical, culturally normative, sequence of events. They challenge individuals’ ability to understand and express themselves as professionals living out an authentic and legitimate career (Ibarra & Barbulescu, 2010). The discomfort provokes individuals to make sense of the disruption and integrate it into a revised self-narrative. While people revise their narratives in the hope of crafting one that is desirable, one that is merely plausible can provide enough meaning to let the person move forward from a disruptive event (Gabriel et al., 2010). Interestingly, the self-narratives people construct from disruptive events can lead to increased professional engagement when they emphasize growth, for instance when setbacks lead people to engage in more deliberate sensemaking and proactivity in their careers (Kutscher & Mayrhofer, 2023; Vough & Caza, 2017). Such narratives can endure and serve individuals as a resource, guiding and legitimating their actions in their daily work (McGivern, Currie, Ferlie, Fitzgerald, & Waring, 2015).

A narrative lens helps us understand how professionals make sense of their participation in collective action in crisis and what it means for their professional roles in the present. First, it directs our attention: experiences within a crisis response gain significance when they support or challenge meanings central to professionals’ role identities (Pratt et al., 2006). Second, it accounts for the ambivalence of crises. It suggests that people will selectively pick out and reinterpret disparate events in the crisis to generate a plausible, coherent, and legitimate story (Ibarra & Barbulescu, 2010). Over time, complex events may be reinterpreted into coherent stories emphasizing themes that direct how people carry out their professional roles in the post-crisis stage (Wright et al., 2012). To understand the self-narratives that emerge from professionals’ collective action in crisis, however, requires us to extend existing theory. Being collective yet temporary events, collective action in crisis represents an experience that differs from both personal setbacks and macro-level change. Unlike the case of institutional changes or social movements, acute crises leave professionals returning to work without a template for role change. Yet, unlike personal setbacks, they see professionals going through shared experiences and so are likely to encourage collective sensemaking (Weick, 1995). Understanding how participants devise stories of professional identity growth from collective action in crisis is the focus of our study.

## Research Context

### SARS in Toronto

Severe acute respiratory syndrome (SARS) emerged in China in 2002 and spread to five continents in 2003. It affected over 8,000 people and caused some 700 to 900 deaths (Skowronski et al., 2005). Canada saw tens of thousands quarantined and 438 cases, including 44 deaths. Almost all occurred in Toronto, Ontario. When SARS struck, the virus was unknown to the medical community. After its arrival with a returning traveller, SARS spread through hospitals infecting patients, visitors and staff through a then-unidentified mode of transmission.

The Toronto outbreak is an ideal case to study growth from collective action in crisis. First, it undermined prevailing ideas in the healthcare sector that infectious diseases no longer threatened developed countries. The initial response (in February to May) took on an improvised character within Toronto hospitals, a process which one interviewee described as ‘building the ship while it was in the middle of the water’ (Ronald,^1^ SARS Scientific Advisory Committee). The response became more systematic during the second wave leading to the end of the outbreak (May to July). The response enables us to explore professionals’ participation in a collective response and subsequent professional growth. Archival materials offer real-time or near-real-time data on this acute stage of response. Second, in the years after the outbreak, participants’ retrospections reveal the contrasting outcomes the crisis had for them. Thus, the time passed since SARS gives us a longer timeline to explore the professional implications of collective action.

### Data collection

We draw on a dataset which the first author began collecting in 2014, 11 years after the outbreak (Table 1). At the time, SARS was unique, the first pandemic threat of the 21st century. We set out to explore collective responses during the crisis. We later shifted our focus to the post-crisis implications, building on a code (‘growth from SARS’) that appeared in the data but which had been peripheral to our original research question. In shifting focus, we turned the time delay into an asset to explore what meaning participants had drawn after the crisis had settled.

**Table 1.** Summary of Case Data.

***Interview***	Semi-structured	37 participants 3 follow-up 11 email follow-up	Hospitals Medical trainees, staff physicians, nursing management, senior management Other settings Local public health, Scientific Advisory Committee, community care organization, medical association
	Informational	8 participants 2 follow-up 2 email follow-up	Expert panel member, medical historian, infectious diseases expert, medical resident, staff physician, chief of staff
***Archival and secondary***	Official reports	3 (Canadian) 2 (WHO)	Campbell Commission, Naylor Report, Walker Report, WHO consensus document
	Presentations	61 public presentations and internal presentations	Presentations from hospitals, professional associations, public health and emergency managers, interest groups
	Memoirs, books, articles	—	Memoirs of participants, medical publications
	Contemporary communications	—	Research, online communications (Pro-MED), media interviews
	Organizational documents	—	Meeting minutes, organization charts, floorplans, annual reports, internal reports and presentations, emails
	Miscellaneous	—	News footage, documentaries
***Other***	Site visits	—	Visits to 4 academic hospitals; community care organization
	Artifacts	—	Personal protective equipment, SARS screening tool, patient transfer forms (blank)

During preliminary data collection, we acquainted ourselves with the outbreak and available data. In 2014 we conducted informational interviews with eight SARS experts, identified through reports and news articles. These participants provided us context and advised us on the technical, medical and legal issues of the outbreak. The archival and secondary data include reports commissioned by the Canadian government and the WHO, 61 presentations by organizations involved, hospital documents, and emails, news footage and memoirs. Table 2 provides identifier codes for sources cited in our findings.

**Table 2.** Document Identifier Codes for Archival and Secondary Sources Cited in Findings.

Document Code	Source
**R-CC**	Report – Campbell (2006).
**IP-MC**	Internal presentation – McGeer (2003).
**MP-BOOV**	Medical publication – Borgundvaag et al. (2004).
**MP-CH**	Medical publication – Cheung (2003).
**MP-MALA**	Medical publication – Maunder et al. (2006).
**MP-PE**	Medical publication – Penciner (2004).
**MP-SCRE**	Medical publication – Schull and Redelmeier (2003).
**MP-STWI**	Medical publication – Straus et al. (2004).
**MP-WE**	Medical publication – Webster (2020).

We conducted semi-structured interviews in 2015–16 with 37 more participants involved in the outbreak and acquired archival data from them, including presentations, photos and meeting notes. We focused on four Toronto hospitals central to the outbreak – given the pseudonyms Downtown, Academic, City and Suburban – as part of a sampling strategy to capture professionals’ response in its fullest form. Staff at other hospitals had less work since many operations were cancelled and suspected SARS cases were transferred to the core hospitals.

Our interviews cover the junior medical ranks (trainees), staff physicians and senior management, and departments pertinent to the outbreak (e.g. emergency and infection control) each in at least two core hospitals, including people who left their organizations post-SARS. We also interviewed people in other healthcare institutions whom participants recommended. Tables 3 and 4 show our interviews across the hospitals and ancillary organizations, respectively. While the time delay between crisis and interview let us see professional growth, it also meant that some participants were unavailable. As it was not our goal to document the containment itself, nor to get a representative sample, we did not consider this a validity threat.

**Table 3.** Core Hospital Interview Coverage*.

Unit	Downtown hospital	Academic hospital	City hospital	Suburban hospital
**ICU**	2	—	—	1
**Emergency**	3	2	—	—
**SARS unit**	—	5	—	1
**Infectious diseases**	5	3	1	1
**Infection control**	1	—	1	—
**SARS management**	3	—	2	3
**Senior management**	3	1	1	1
**Other units**	1 (psychiatry)	2 (research)	1 (family) 1 (research)	1 (family) 2 (surgery)

* Participants occupying multiple positions listed more than once.

**Table 4.** Interview Coverage in Ancillary Organizations.

Position	Local Public Health	Scientific Advisory	Community Health	Medical Association	Community Hospital
Sr. management	1	1	2	2	—
Other	1	4	—	—	1

The interviews ranged from about 30 minutes to 1½ hours, were conducted in person, by telephone or online. We asked participants to describe their duties prior to the crisis, how their duties changed during the acute stage, and their reflections post-crisis. To avoid validity threats associated with retrospective interviews (McCracken, 1988), we distinguished between data about the acute crisis (events in 2003) and the post-crisis phase (meanings attached to SARS 2014–16). We treated the latter as an outcome of sensemaking, narratives guiding participants’ careers independent of whether the memories had been distorted or revised (Weick, 1995).

To increase the validity of observations about the acute phase, we used strategies suggested by Miller, Cardinal, and Glick (1997). First, we triangulated critical incidents with archival and secondary sources, much of which had been produced by participants themselves in articles, interviews and investigations closer in time to the outbreak. Second, we sought corroboration where interviewees’ stories interconnected. Third, we relied on participants to point us to people or documents which we used to verify facts.

### Analysis

We began open coding and memoing to interpret our interviews and archival and secondary data (Glaser & Strauss, 2009). In this first stage we categorized incidences of staff participating in the collective response to SARS within or through their hospital. We conceptualized participation as a form of temporary role adaptation in an established organization. We counted as role adaptation any time that (a) a member was assigned or requested by a superior to a SARS-related task, (b) volunteered to a call for help for such tasks, or (c) invented a role for themselves in their hospital’s response. Coding was mostly straightforward, as the role was clearly related – in the minds of the person and their organization – as a response to SARS.

While official reports emphasized the organizational challenges of containing the outbreak, the interviews we conducted and memoirs we read stressed personal aspects. We developed first- and second-order codes to capture participants’ experience, primarily paraphrasing participants’ words (e.g. many used ‘bearing the burden’ to describe patient care). SARS was a difficult event. Some had tried to forget the whole thing, finding their involvement unappreciated and withdrawing from positions that would expose them to future outbreaks. To our surprise, other participants expressed that SARS had been a positive professional experience, something that had inspired or even benefitted their careers. We moved to a second stage of analysis to understand how participants derived these contrasting meanings.

In the second stage, we compared and contrasted instances of role adaptation and found them associated with different meanings.^2^ One type of role was associated with seeing SARS as an event lacking professional relevance (what we labelled ‘idiosyncratic assignments’). Another role was associated with inspiring participants’ professional identities (‘deployment roles’). A third role was associated with tangible career rewards (‘steering roles’). Previous studies had argued that professionals’ involvement in collective response could inform their subsequent role change (Compagni et al., 2024). But they did not explain the variance in our participants’ stories, nor their divergent professional engagement with the crisis afterward. To explore these associations, we turned to the literature on professional identity narratives (Ibarra & Barbulescu, 2010).

**Figure 1.**
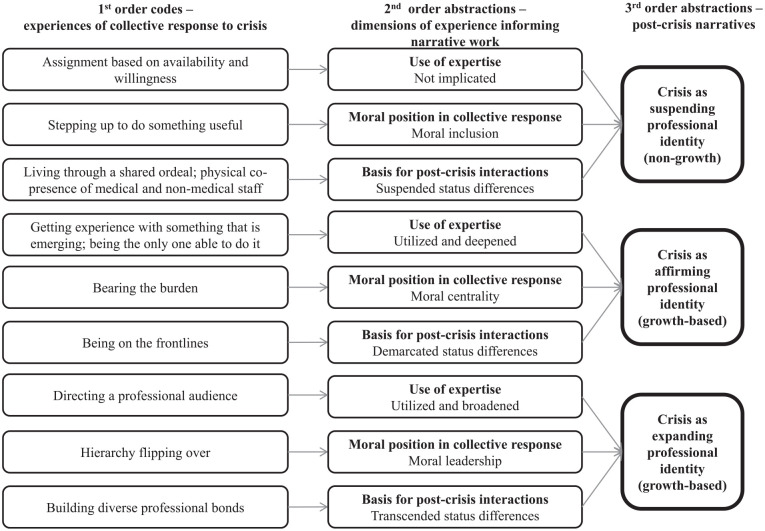
Data structure.

In the third stage, we analysed how one’s actions during the crisis might inform post-crisis narrative work and subsequent professional growth. We theorized that the three types of role adaptation afforded different opportunities for hospital staff to (1) receive cues from colleagues about the expert status and moral significance of their chosen professional specialism and (2) engage in follow-on interactions with the colleagues with whom they had worked to relate these meanings to future professional role change. Figure 1 presents our data structure. Finally, through additional analysis we refined our findings to develop our model and account for counter-examples and people acquiring multiple roles in the crisis.

## Professional Growth from Collective Response to Crisis

**Figure 2.**
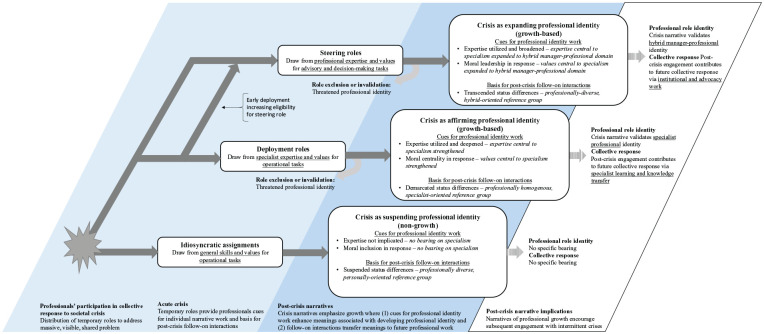
Stories of professional identity growth from collective action in crisis.

Years after the outbreak, an investigation of the Ontario healthcare sector quipped ‘Starting with nothing . . . this jerry-built apparatus somehow did stop SARS’ (R-CC, p. 355). Within and across hospitals in Toronto, staff ranging from medical trainees to department chiefs to accountants had put together a collective response to contain the outbreak. The experience was challenging and, for some, traumatic. Yet, many had surprisingly positive stories about SARS, seeing it as a time where they had grown as medical professionals. We trace these divergent stories to the division of labour in hospitals’ collective response. The response involved a variety of temporary roles which staff volunteered for, were assigned to, or self-initiated. We present SARS as it was experienced through three roles in the response – *idiosyncratic assignments*, *deployment roles* and *steering roles* – which were respectively associated with narrating SARS as an event that *suspended*, *affirmed* or *expanded* one’s professional identity. Only the latter two roles were associated with growth and continued inspiration for how participants enacted their professional roles post-crisis. As Figure 2 illustrates, we attribute the difference to deployment and steering roles (1) drawing participants into interactions within the collective that enhanced meanings central to their professional identity (in the acute crisis) and (2) led to follow-on interactions that provided a platform to translate these meanings into professional role change (post-crisis). Together, these elements let the person narrate the crisis as growth, a sequence of events which drew from their earlier professional self and contributed to their future professional self.

### Idiosyncratic assignments: Crisis as suspending professional identity

Idiosyncratic assignments describe staff who were called to, volunteered for, or initiated operational roles in their hospital’s SARS response that did not draw from their professional specialization. Despite being a medical emergency, much of the effort drew only on members’ general skills. For instance, emergency measures required people entering hospitals to be screened for respiratory disease. Non-medical staff volunteered alongside nurses for this role. Moreover, idiosyncratic assignments involved operational-level tasks, such as procuring equipment rather than managing other professionals. Overall, idiosyncratic assignments could inspire staff to have a sense of contributing in the crisis, and even that something about their character had been revealed. Yet, they did not encourage stories about professional growth.

#### Expertise not implicated

The tasks involved did not require specialist expertise and deemphasized staff’s usual role in the hospital. As Gemma (ICU physician, Suburban) explained, idiosyncratic assignments were assigned based on *availability and willingness*: ‘People wanted to help us . . . We could find roles for just about anybody, even if they had no idea, never been in an ICU before, had no idea how to look after [patients], we could find something for them to do.’ Thus, participants talking about idiosyncratic assignments emphasized how much the tasks contrasted with everyday work. For example, Nikolai (surgeon, Suburban) described the surreal experience of being put in charge of procuring equipment for his hospital: ‘I was given a cart blanche for ordering supplies. I was spending hundreds of thousands of dollars . . . without needing approval from top management.’

#### Moral inclusion in the collective response

Because expertise was unnecessary, idiosyncratic assignments gave staff opportunities to be included within the collective moral effort, independent of their profession. Staff talked about *stepping up to do something useful*. This was attractive, as most physicians had surprisingly little to do in the crisis. Andrew (ICU physician, Downtown) explained: ‘It was the most incredibly boring time because the hospital was dead quiet. Everything was closed.’ Instead of seeing patients, physicians engaged themselves in non-medical roles, like buying equipment for makeshift wards or communicating with the media or physician associations (R-CC). Gemma (ICU physician, Suburban) worked with a nursing manager to procure equipment for a SARS ward, a role that gave her a sense of contributing: ‘We wanted to be there and we could help and it was important work.’ She sees the experience as ‘positive’:

I think the whole experience was positive, believe it or not. I recognize it was terrible for lots of people, but I think in many ways it was very positive for our hospital. It was positive for me personally because I felt like I had gotten through it and I was strong through it and survived it.

#### Suspended status differences

In deemphasizing expertise, idiosyncratic assignments brought staff from diverse professions together and suspended the hierarchies governing hospital life. What mattered was who was willing to show up rather than their rank. Participants felt that they were *living through a shared ordeal*. With most staff evacuated from the buildings, individuals like Nikolai (surgeon, Suburban) who turned up described newfound ‘respect for housekeeping and social workers’ who remained at the hospital, as did Alex (medical trainee, Downtown) who was inspired to see non-medical staff ‘setting an example through the sheer act of showing up’ even though ‘they had not taken a professional oath’. Idiosyncratic assignments further fostered solidarity through *physical co-presence of medical and non-medical staff*. A common sight was seeing diverse occupations donning identical gear and working alongside. Tara (nurse, Downtown) describes the solidarity among them:

[There was a sense that] ‘We were the few’ . . .. A lot of people bonded in some unusual ways, because people who normally did risk management or who managed the IT infrastructure had all been pulled from their posts to screen staff and patients at the front doors. So everyone was reassigned roles . . . it was a kind of a ‘lock arms and close the gates’ mentality.

#### Emerging narrative: SARS as suspending professional identity (non-growth)

For participants reflecting years after SARS, idiosyncratic assignments lacked the sense of growth we found in other roles. In the language of coping, staff were telling stories that discounted growth from SARS – emphasizing instead the extraordinariness of the event or how they survived it (cf. Vough & Caza, 2017). Participants remarked how joining in the response could suspend meanings associated with being a medical professional, such as expertise or status. But routine had resumed after the crisis. SARS might be something to look back upon with pride, fear or resentment, but it was an aberration rather than a step along in one’s professional development. As Figure 2 illustrates, we attribute these narratives of non-growth to (1) the lack of relation between idiosyncratic assignments and core meanings associated with participants’ professional identities and (2) lack of follow-on collegial interactions that could help participants relate their experiences to post-crisis professional role change.

First, because idiosyncratic assignments rely on generalist skills and values, they do not evoke meanings directly related to one’s professional identity. Participants found it hard to integrate their tasks into a story about developing professionally. For instance, when discussing the non-nursing roles she had taken, Tara (nurse, Downtown) struggled to identify skills that could be applied to her profession: ‘I felt that I had gained some sense of personal resilience, but specific skills, I’m having trouble thinking of something.’ Likewise, the operational-level work did not draw participants into interactions that evoked their professional values. Even Gemma (ICU physician, Suburban), despite feeling positive about her contribution, acknowledged that it had little significance for her medical values: ‘[my immediate colleagues] were very supportive. . . But it was a small number. I think the general population had absolutely no idea what we were going through.’

Second, while idiosyncratic assignments can suspend status differences, they do not encourage follow-on interactions post-crisis that translate participants’ experiences into subsequent professional role change. Diverse staff brought together around non-specialist tasks lack a shared basis to continue interacting professionally once the crisis has settled. Participants focused instead on the personal friendships they had made. Those who had worked alongside each other spoke about meeting post-SARS for coffee or dinner, where they might reminisce, express mutual admiration, or joke about seeing each other maskless for the first time. Gemma, the ICU physician, remarked that she had become ‘very good friends’ with the nurse she had worked alongside but acknowledged that they saw each other at the hospital ‘only occasionally’.

Ultimately, a narrative about the crisis suspending one’s identity seems to provide no specific guidance for professional role change. Thus, we argue that idiosyncratic assignments are unlikely to encourage continued professional engagement with the crisis topic that would either impact daily professional role enactment or build capacity for collective responses to future crises. However, we identified other roles staff that undertook during the collective response to SARS which, as we explain next, led them to see the crisis through the lens of professional identity growth.

### Deployment roles: Crisis as affirming professional identity

Deployment describes staff pulled into the operational roles of containing SARS in ways that drew from and developed their professional specialism. This includes the physicians and nurses who worked on the SARS wards. Deployment let individuals feel they had made a unique and essential contribution within the collective response, both as an expert and as someone bearing moral responsibility. It also provided a basis for follow-on interactions through which staff reflected on, and created lessons for, their future professional practice. Participants spoke of SARS in terms of growth: as an affirmation of their professional identities – something that validated, clarified or enhanced meanings associated with their specialism.

#### Expertise utilized and deepened

Deployment drew on participants’ specialist expertise, demonstrating and deepening its perceived value. These roles gave an exclusive group of staff – mostly physicians but also some medical trainees – the experience of being part of a select few in their field who were *getting experience with something emerging*. Medically, SARS was exciting for those who had dedicated their careers to infectious diseases (ID). Before 2003, coronaviruses such as SARS had been associated with mild illnesses, considered ‘uninteresting’ and studied by people in ‘small basement offices’ (IP-MC). Then SARS thrust Toronto’s ID specialists to front stage. Marya (ID specialist, Academic) expressed excitement about deploying to treat SARS, adding that it affirmed one of the ‘great things’ about her specialism:

One of the things I felt about SARS was I felt very energized . . . Although it was stressful and it was hard, it was an opportunity. And that’s one of the great things about infectious diseases, that new things come along out of the blue . . . when something new comes along, something you’re not familiar with, and you haven’t seen it before, you get very energized. As a physician, I think it was part of that excitement.

Relatedly, colleagues’ requests for help signalled to participants that their expertise was uniquely valuable in the crisis, creating the experience of *being the only one able to do it*. ‘SARS was the opportunity of a lifetime’ remarked Anna (ID specialist and postdoc, Downtown) who volunteered on a SARS ward at Academic Hospital. ‘There were not a lot of infectious diseases people . . . they needed my help because there was a staff shortage.’ Being the only one eligible for deployment could make individuals see the significance of their specialism, like Danielle (medical trainee, Downtown): ‘I felt I could [reassure worried colleagues] no problem and in a genuine way because I had my personal experience [with SARS].’

#### Moral centrality in the collective response

Exclusivity provided a sense of being morally central, something participants associated with proving their professional values. Staff deploying felt they were part of the few *bearing the burden* within the collective effort. To reduce disease transmission, hospital policies encouraged a small group of staff to specialize in treating patients. While deployment exposed staff to the virus, reports afterward surprisingly concluded that it did not lead to worse psychological outcomes (MP-MALA). Henry (ER physician, Downtown) believes that the hardship was offset by the sense among those deploying of making a ‘particular contribution’:

There were two or three areas in the hospital that were intimately involved in the care of SARS patients . . . In other units that were at far less risk the anxiety and absenteeism [and] the need for attention was much greater . . . if you think about the things that I said gave our staff courage, the people [in other units] not directly involved weren’t being recognized for any particular contribution because they were tangential to the main fight.

#### Demarcated status differences

In combining expertise with moral centrality, deployment roles created an insider–outsider boundary. There were those *on the front lines*, a professionally homogeneous group of physicians and nurses treating patients, and there was everyone else. The hardship encouraged participants on the wards to contrast themselves with colleagues who refused deployment, avoided the hospital or sheltered in offices. Two physicians reflected: ‘The red indentations [from protective gear] across our faces at the end of each day seemed vaguely honourable . . . [but] not all physicians, nurses and support staff were willing to do front-line SARS care’ (MP-SCRE, pp. 122–123). Staff could feel alienated from colleagues who did not see patients. Marya emphasized that the credit for containing SARS had gone to those ‘higher up . . . none of whom actually saw a patient, none of whom actually had put themselves at risk’. Thus, while solidarity emerged among those deploying together, it led to sharper distinctions between those who lived up to their medical oaths and those who did not.

#### Emerging narrative: SARS as affirming professional identity (growth-based)

Years afterward, deployment could lead individuals to see that the SARS crisis had been an affirmation of professional identity. They told stories about SARS as a sequence of events through which they had developed as professionals, taking the form of a growth-based narrative (cf. Vough & Caza, 2017). Participants could see SARS as a time where their expertise deepened, where their professional values were proven, and where they clarified their career interests. As Figure 2 illustrates, we attribute these narratives of identity affirmation to (1) deployment roles *strengthening* meanings central to one’s professional specialism and (2) the potential they created for follow-on interactions helping participants relate these meanings to post-crisis *specialist* work.

First, participants found it easy to integrate SARS into a story about developing as medical professionals. They had seen the relevance of their expertise in a crisis and the demand it created for their involvement. Likewise, moral centrality with one’s co-specialists elevated the worth of the specialism, renewing participants’ sense of purpose. SARS became the inspiration for subsequent projects. Richard (ICU physician, Academic) was among those who shifted his research interests to ID after deploying on a SARS ward, ‘I became specifically interested in how to speed up research during outbreaks, so the information could come in sooner and help manage the outbreak itself.’ Physicians also reported that deployment affirmed their fit with medical values, believing that they had been given the chance to ‘role model their commitment for the [trainees]. As part of being a doctor you need to be there on the front lines’ (MP-2, p. 84). Living up to such values could clarify for those earlier in their careers that the specialism was right for them. Jennifer (medical trainee, Downtown) was inspired to focus on public health. Years later, she had integrated the SARS and Ebola outbreaks into a narrative about professional growth:

Having gone through it twice, first with SARS, then with Ebola . . . if you’re involved in something that’s emerging, you grow in the competency of a leadership role a lot faster, and you’re often put in above where you’d be . . . For me as a person, [SARS] was both horrible and professionally very interesting at the same time. I never talk about it because these are things that don’t sound pretty . . . But it’s true. It was probably one of the biggest defining points for me during my residency training. It certainly pushed my interest in public health.

Second, by demarcating status differences, deployment encouraged follow-on interactions that helped participants relate the crisis to their future specialist practice. Deployment brought together a homogeneous group of professionals sharing interests in the medical and scientific aspects of SARS. The bonds developed among co-specialists on SARS, ICU and emergency wards provided a platform for projects such as research papers and reflection pieces through which participants consolidated and claimed new specialist knowledge (MP-BOOV; MP-SCRE). Yet, while participants felt they had become better professionals, they did not consistently see SARS as an opportunity (MP-STWI).^3^ Many remarked that their operational-level tasks lacked visibility (R-CC, p. 557]) and described themselves as ‘grunt[s]’ (Marya, ID specialist, Downtown) or ‘unsung heroes’ (MP-CH, p. 1285) in reflection pieces they wrote for each other. Even the professionally inspired Jennifer saw few tangible rewards: ‘I didn’t need credit . . . of course we were invisible.’ Invisibility created ambivalence. Individuals felt pride in their profession but also devalued within the healthcare system. Vera (nursing manager, Downtown) explained, ‘Nurses were invisible to the public, highly trusted, but invisible.’^4^

The counterfactual of these affirmation stories was the sense that the crisis had threatened one’s professional identity. Even here we find that deployment, or exclusion from it, encouraged participants to see continuity between SARS and their identities afterward. Negative experiences could lead people to disengage professionally (R-CC, p. 979, 990). For instance, most medical trainees were prohibited from entering hospitals for their safety. Dana (ID specialist, Downtown), who helped devise the policy, recounted the outrage trainees had about being excluded: ‘They were really angry, because they were trying to be doctors and they were there to help patients and we specifically excluded them.’ Likewise, because participants saw deployment as an enactment of values, poor experiences could challenge their sense of fitting the profession. Winona (ID specialist, Community Hospital), who found deploying on a SARS ward traumatic, drew the lesson that the specialism was not for her: ‘After SARS I switched to slow diseases . . . I feel better now but I’m not returning to the field anytime soon.’

Ultimately, by affirming professional identities, we argue that deployment is likely to encourage subsequent specialist engagement with topics relating to the crisis. As identity research shows, people are motivated to pursue aspects of their identities that provide affirmation (Pratt et al., 2006). Participants deploying could feel their specialism had been affirmed by the crisis. We found them engaging in specialist learning and knowledge transfer activities which years later would contribute to their profession’s response to Ebola and Zika. Trainee Jennifer – who had unclear career interests pre-SARS – emerged with a story articulating her interest in global health, one she took to fighting Ebola a decade later. We likewise saw Richard who, inspired by his experience, went on to conduct pandemic-related research. Moreover, these narratives embodied the moral values that guide professionals’ responses in crisis (Wright et al., 2021). Participants linked SARS to their proactivity in protecting colleagues (Henry) or advocating for patient treatments (Sophia) during Ebola (Table 5 in the Appendix). Yet, despite the inner growth that deployment fostered in professionals, it provided little external recognition of participants’ contribution in the crisis. In the next section, we examine staff who undertook a third type of role associated with more tangible career opportunities.

### Steering roles: Crisis as expanding professional identity

Steering roles describe staff pulled into expert advisory and decision-making roles during the crisis. This includes members invited by hospital management to SARS committees whose members developed organization-wide policies, set up screening centres and organized SARS wards. These roles drew on one’s professional specialism, just like deployment. But steering roles went further. They entailed a broadening of professional specialism and wider peer validation of one’s contributions, providing a basis for follow-on collaborations at the managerial, institutional or field level. Reflecting on steering, individuals spoke of SARS as an opportunity for professional identity expansion: an event that provided the resources for them to broaden from their specialist role identity into new domains within the professional field.

#### Expertise utilized and broadened

Steering roles drew on participants’ specialist expertise for extraordinary and highly visible tasks in the collective response. Participants found themselves broadening their repertoire of professional skills, for instance using their specialist expertise to advise on hospital management and provincial policy. Unlike deployment, where expertise might be invisible, steering roles made one’s colleagues recipients of that expertise. Those in steering roles found themselves *directing a professional audience*, sharing their knowledge in assemblies ranging from small groups to hospital-wide gatherings. Given the urgency of SARS, authority sometimes migrated to specialists such as infection control staff. Bernard (infection control, City) recounts how he came to co-steer the hospital’s operations:

I did a really crazy thing – I actually called the hospital CEO . . . you could imagine [if his response was] ‘look at this 30-year-old-punk who just came out of school, why am I listening to you?’ . . . it could’ve gone very badly. [*The CEO instead called him to a meeting*] . . . I walk into this meeting . . . And the CEO just says, ‘Okay Bernard, tell everybody what’s going on . . . what do you want us to do? . . . My role in all of that was, making the call early on for our hospital, and then I actually lived in the CEO’s office for months. I went there every day, sat there with CEO. We had a SARS emergency ops centre which was just behind his office.

#### Moral leadership in the collective response

From colleagues’ feedback, junior physicians and staff specialists felt that their expertise gave them the power and responsibility to exercise leadership within the collective. For the first time in their careers, senior physicians and managers were seeking their guidance. In extreme cases, participants experienced the *hierarchy flipping over*. Since the virus was new, junior staff who treated patients early on obtained experience that their senior colleagues lacked. The deference afforded to trainees like Helen (Downtown) and Anna (City and Suburban) was extraordinary as they were invited into steering roles because they had been early deployers. Helen (medical trainee, Downtown) recounted being elevated as an authority by colleagues who requested her to give a hospital-wide lecture:

I put together a slide deck to teach, in this huge auditorium, all this staff, which again was this whole hierarchy thing being flipped on its head, where they turned to me and said ‘You’re the expert, you tell us what we need to do.’ And I just remember looking into the audience and thinking ‘but you guys are still teaching me, and you’ve made that very clear. You guys teach me, I don’t teach you. That’s been the message all along. All of a sudden now I’m the expert?’

Helen afterwards found herself being approached by senior physicians requesting consultation on PPE use, patient management, and even whether Downtown Hospital should be shut down. As Helen describes, the moral leadership thrust upon her was both empowering and disorienting, ‘[the senior physicians] were so respectful of the knowledge that I was imparting to them . . . [but] that’s not how the hierarchy works, right?’ While the hierarchy still existed during SARS, those in steering roles felt that they had been decoupled from it, acting as an expert and moral leader unconstrained by rank.

#### Transcended status differences

Participants remarked that their expert and moral contribution was professionally recognized in the collective, letting them transcend the status associated with their junior or specialist position. As for the path to recognition, participants singled out participation in SARS committees as occasions for *building diverse professional bonds*. Such committee work entailed collaboration with high-ranking colleagues within and beyond their organization whom they otherwise would not have met, creating an opportunity to become network brokers (cf. Burt, 2004). Anna (medical trainee) volunteered to treat patients at Academic and Suburban, later being invited to the SARS committees of both, where she ‘felt a great bond with the colleagues I worked with, like [senior physician] and [future CEO of Suburban] . . . fighting SARS was the opportunity of a lifetime.’

#### Emerging narrative: SARS as expanding professional identity (growth-based)

Years afterward, steering roles could lead individuals to talk about SARS as an event that had fast-tracked their careers and expanded their identity beyond the confines of professional specialism to someone who was a leader or advisor in their field. Participants emphasized that SARS had brought inner growth along with tangible benefits that further established their reputations. As Figure 2 illustrates, we attribute these narratives of expanded professional identity to (1) steering roles *expanding* meanings central to one’s specialism and (2) the potential they created for follow-on collegial interactions to relate these meanings to post-crisis *hybrid manager-professional* work.

First, steering roles utilized and broadened participants’ professional skills to new and visible tasks. This encouraged them to see their specialism as a springboard for activities such as advisory work post-crisis. Participants could likewise point to experiences of moral leadership entrusted to them as something increasing their confidence in interpersonal tasks such as hospital leadership, advocacy work and consulting gigs. Bernard (infection control, City) believed his steering role ‘launched [his] whole career’ and enabled him to do healthcare consulting:

In terms of my career I was just starting out. It introduced me to doing media interviews. It introduced me to every senior leader in the hospital, and to this day my role in the hospital is so much more prominent than it should be because of SARS . . . it launched my whole career. Just being in the right place at the right time . . . I have the street cred to come in through the door [of other organizations] . . . they know at least I’ve dealt with SARS and done so much media in addition to traditional academic jobs.

Second, the transcended status differences – consisting of diverse, professional connections – supported narratives emphasizing professional identity expansion with tangible, follow-on career opportunities. Participants’ demonstration of expertise and leadership competence, along with new connections to senior healthcare managers, put them in an ideal position to get invited to high-impact professional projects in the years after SARS. The committee members Anna (medical trainee) got to know at Suburban invited to co-author research and referred her to national media which featured her as a ‘hero’. In contrast to the more specialist-oriented projects pursued by those who had deployed, participants associated steering roles with projects relating to institutional leadership and advocacy, such as provincial inquiries on pandemic preparedness. These projects provided a basis for ongoing high-level professional collaboration post-SARS and further visibility of one’s professional competence, encouraging participants to see that they had come out of SARS with a fast-tracked or expanded career in healthcare.

Ultimately, by inspiring professional identity expansion, we argue that steering roles are likely to encourage subsequent hybrid manager-professional engagement with topics related to the crisis. Research shows that positive experiences of managing other professionals can encourage professionals to embrace a ‘hybrid’ outlook that emphasizes managerial solutions to problems, for instance physicians redefining healthcare from an individual activity (e.g. valuing clinical practice) to a collective one (e.g. valuing management in healthcare delivery) (McGivern et al., 2015). Participants who had steering roles could feel that their professional specialism had been expanded during SARS, becoming a springboard for advisory and decision-making roles. We found them engaging in institutional and healthcare advocacy work which years later would contribute to their profession’s response to future pandemics. Physicians who took on steering roles, like Bernard and David (see Table 5 in the Appendix), went on to seek consulting opportunities, to advocate for pandemic preparedness or to establish peer networks, developing the institutional structures that hospitals would draw on to manage future outbreaks years later (MP-WE).

Even in the counterfactual, when things went poorly, steering roles implicated participants’ professional advancement. As visibility could earn one credit for perceived successes, so could it afford blame for failures. Timothy (ICU physician, Downtown) was one of several participants who contrasted the fates of two leaders in provincial SARS committees, one who was elevated as a ‘hero’ and another ‘demonized’ by his colleagues and the media. In both cases, the narrative that emerged was one of opportunity: SARS could make careers, and it could break them.

## Discussion

We set out to explore how professionals develop narratives of growth from collective action in societal crises, which involves undertaking tasks ranging in expertise-use and operational level within a distribution of temporary roles. We identified two elements of participating within a collective response that encourage stories of growth. First, performing the role enhances meanings central to the individual’s professional identity, particularly from collegial feedback on their specialist expertise and values. Second, after the crisis, it facilitates follow-on collegial interactions that provide a platform to turn these meanings into post-crisis professional role change. Together, these elements let the professional narrate the crisis as growth, a plausible sequence of events which both draws from their earlier professional self and contributes to their future professional self. Our narrative explanation contributes to the literature on collective action by accounting for the variance and endurance of professional role change among participants post-crisis. We also extend theory about the fragmentation of perspectives in crisis response, showing how it can continue into the post-crisis period and affect further professional collaboration. Finally, we contribute to the literature on narrative identity work by highlighting how follow-on collegial interactions arising from collective disruptions can direct individuals towards different professional growth paths.

### Explaining professional growth and role change after collective action

By examining professional identity growth, we contribute to the literature on professionals’ collective action in crisis (Beck & Plowman, 2014; Jiang, 2021; Kornberger et al., 2019; Wright et al., 2021). Whether professionals grow from collective action in crisis is a significant issue as it is likely to impact both their own careers and their field’s capacity for collective action in future crises (Dwyer & Hardy, 2016). However, limited empirical research to date has made it difficult to see the extent that professionals’ experiences inform their work in the years post-crisis (Compagni et al., 2024, p. 857), especially when collective action in acute crises does not provide participants with a template for role change.

Taking a narrative approach, we found participants telling different stories about whether and how collective action inspired their professional growth in the years post-crisis. Figure 2 proposes that growth depends on insights being captured and integrated into the self-narratives professionals create around the crisis. Professionals use self-narratives not only to convey but to construct and enact their identities (Ibarra & Barbulescu, 2010). When professionals recollect, tell or refer to their crisis narratives later in their careers, they are likely to remember and give weight to those events consistent with the story’s themes and sentiments. The implication is that professional growth post-crisis will depend on the extent to which new practices and perspectives are integrated into narratives that inform professionals’ role enactment.

Our study presents two main implications for understanding post-crisis professional role change. First, we show that the extent to which professionals implement the practices and perspectives discovered during a crisis into everyday work post-crisis depends on a supporting narrative. We found many participants who associated joining in the collective action with positive emotions but who nevertheless saw the crisis as irrelevant to their subsequent professional work. These were participants who had narratives of identity suspension. In contrast, those with growth narratives continued engaging with practices and perspectives they had discovered from the crisis. The difference was that these professionals had made them a part of the story of their ongoing professional growth. Thus, while our findings fit the idea that emotions can drive organizational and institutional change (Zietsma & Toubiana, 2018), they also highlight the importance of professional identity narratives that link emotions associated with a crisis to professionals’ continued engagement with that crisis as they settle back into their everyday work.

Our study further emphasizes the varieties of professional growth that can arise among participants involved in the same crisis. In doing so, we go beyond previous research focusing on the shared direction of professional growth among participants post-crisis (Jiang, 2021; Sahay & Dwyer, 2021). For example, Compagni et al. (2024) emphasized how Covid-19 undermined physicians’ expertise, encouraging them to adopt service-oriented practices to continue helping others. In contrast, we found some professionals feeling that their expertise was enhanced by crisis (strengthening their specialist identity), with others seeing value in non-specialist practices (expanding their specialist identity into a hybrid manager-professional identity). Enduring appreciation for new practices seems to depend on their fit with professional identities emerging from the crisis. Thus, specialists with stories of identity affirmation, even if they appreciate service-oriented practices, may not be motivated to continue with them. Instead, their efforts may be directed at honing the technical expertise they gained in the crisis. Overall, we propose that professional identity narratives act as a filter, guiding which of the many adaptations professionals make during a crisis continue to be seen as valuable to their post-crisis work in the role of a specialist, hybrid manager-professional, or other type of professional.

### Extending the fragmentation perspective to collaboration between intermittent crises

By exploring the divergent stories professionals tell about growth, we extend scholars’ understanding of the fragmentation of perspectives that arise in crisis response (Wolbers et al., 2018). Crisis response among distributed actors requires participants to coordinate in some form to achieve shared goals (Beck & Plowman, 2014; Kornberger et al., 2019). Nevertheless, as Wolbers et al. (2018) show, crisis response among actors distributed within and across organizations also tends to generate a fragmentation of perspectives. The idea is that responding to a novel, urgent problem requires participants to work around procedures, delegate tasks and claim expertise in ambiguous situations, which foil shared understandings such as hierarchies of control or planned procedures. These practices address urgent problems – for instance, by letting front-line responders provide shelter for people in an emergency without having to wait for formal approval – but generate a multiplicity of perspectives about what is going on in the crisis across operational levels. While Wolbers et al. (2018) focus on coordination in one-off emergencies, our study draws out the implications that fragmented perspectives can have on professional collaborations between episodes of intermittent crises.

A key implication is that the distribution of roles within a response can generate a morally charged fragmentation of perspectives that then plays out into post-crisis professional collaboration. As Figure 2 outlines, collective responses put participants into roles that differ across multiple dimensions: operational level, moral position and expertise use. This part was not novel, as previous studies establish the division of operational (i.e. front line) versus tactical (i.e. steering) levels in crisis response, differing levels of moral centrality (i.e. direct versus indirect responders) and authority migration (e.g. authority shifting to those closest to the incident) (Bigley & Roberts, 2001; Rapp et al., 2024). What is novel is that we found participants developing fragmented perspectives by looking at their experiences *across* the three dimensions and integrating these into morally laden narratives about what happened in the crisis. Since these dimensions include inputs – how the person contributed during the crisis (expertise-use and moral position) – and outputs – how the person benefited from it (follow-on interactions enabling professional growth), their integration into narratives creates variance in the degree to which participants may feel their contributions were adequately recognized by their professional field.

The fragmented perspectives imply that participants can exit an otherwise successful collaboration with fundamentally different narratives of what the crisis meant, like whether it was a transient event, an underappreciated sacrifice or a career opportunity. Emergent collaboration in crisis appears likely to generate ambivalence among participants undertaking roles with high moral centrality but low task visibility, as was the case with deployment roles. Ambivalence was less common among those who had undertaken idiosyncratic assignments or steering roles. For these individuals, the contribution participants felt they had made (low and high, respectively) seemed consistent with the recognition they received from colleagues (low and high). Grievances, conflicts and misunderstandings may arise where there are contrasts of meaning *within* the narratives of professionals (e.g. a professional’s sense of moral centrality contrasting with lack of follow-on opportunities) or *across* narratives of different groups (e.g. whether moral position was consistent with decision-making opportunities during the crisis). This is significant for the post-crisis stage which is an important time for professionals and their organizations to recover and prepare for future crises, for instance through inquiries, commemorations and development of support networks (Dwyer & Hardy, 2016). When high moral centrality or expertise-use lead participants to become disillusioned about the lack of follow-on opportunities, decision-making involvement or recognition from colleagues, they may withdraw from post-crisis recovery and preparation processes to the detriment of their organizations and professional field.

### Narrative identity work in disruptive events

By highlighting the role of follow-on interactions, we also contribute to the literature on how professionals use narrative work to restore or revise their identities following disruptive events (Gabriel et al., 2010; Ibarra & Barbulescu, 2010). Previous studies show that narrative work can enable post-disruption career growth, especially when narratives encourage deliberate sensemaking about career opportunities, discovery of desired possible selves, and strategies for attaining such selves (Kutscher & Mayrhofer, 2023; Vough & Caza, 2017). Moreover, studies stress that people test out, revise and discard narratives in their repertoire through interaction with audiences such as family, friends and colleagues (Ibarra & Barbulescu, 2010; Kreiner, Hollensbe, & Sheep, 2006). Such audiences hold different expectations about what is a legitimate narrative and can redirect the teller’s story by granting legitimacy, withholding it or filling in elements. Where audiences are supportive, previous research indicates that they increase the likelihood of growth stories (Shepherd & Williams, 2018; Vough & Caza, 2017).

We extend prior research by highlighting a dynamic which becomes important in collective disruptions such as extreme events, layoffs and organizational decline: follow-on interactions that guide participants’ efforts to revise their professional identity narratives. The formation of follow-on interactions means that individuals revise their narratives in a context that differs from the individual setbacks or career transitions that have been the focus of previous studies (Pratt et al., 2006). Our study specifically draws attention to how a participant’s role during a collective disruption entwines them in different follow-on interactions post-crisis that reinforce personal, specialist or generalist perspectives on members’ shared experiences. These interactions pull together colleagues into groups that might not otherwise deeply interact (e.g. spanning across specialisms or levels of hierarchy) and can steer the narrative work of their members towards divergent professional paths.

We see follow-on collegial interactions that emerge post-crisis as having two significant consequences for narrative identity work. First, when interactions pull together professionals with shared experiences and interests, they may help members support each other to construct growth narratives as they are well positioned to validate and fill in elements of a story relating to their professional specialism (Croft et al., 2015). Supportive follow-on interactions may take forms such as debriefings, inquiry focus groups, research projects or support networks. As seen in Figure 2, when a role leads to follow-on interactions with people in the same specialism and a focus on the event from an expertise perspective, we propose that it will support growth narratives valuing a specialist career path. In contrast, when a role leads to follow-on interactions with people in different specialisms but with a shared interest in creating knowledge, it will support a focus on managerial insights and the value of a generalist career path. Thus, professionals going through the same crisis could exit pursuing different career trajectories reinforced by the colleagues with whom the crisis had connected them. A second implication is that follow-on interactions might also lead individuals towards narrowing views of what a crisis meant. While turning to those with similar experiences may be reassuring, it may limit the breadth of participants’ narrative repertoire (Ibarra & Barbulescu, 2010) and leave them less able to empathize with colleagues whose views of the crisis seem increasingly unrelatable. Overall, we suggest that researchers attend to the follow-on collegial interactions – including factors such as membership criteria and common purpose – to understand the varieties of professional self-narratives emerging from collective disruption.

### Limitations and future research

We set out to explore the growth stories of our participants. These stories were to us the most surprising and in the literature least understood. Indeed, interviewees who were eager to talk about their growth reflected the taboo nature of the topic, hesitating to sound eccentric or insensitive. While emphasizing growth then, we recognize that SARS was for many grim, including those who lost colleagues or were physically injured. Future research might build on our study in several ways. First, longitudinal studies might test and elaborate on the implications we drew about post-crisis narratives encouraging professional growth. Studies might trace the scientific, networking or institutional work emerging from post-crisis role change and how it contributes to collective action during acute episodes of intermittent crises. Second, studies might develop our understanding of follow-on interactions following collective events, for instance by exploring different contexts (e.g. mass layoffs) or venues (e.g. commemorations and informal networks) and how they encourage various career narratives following disruption. This work can build on previous studies that have observed that post-disaster processes like inquiries and commemorations engage participants around different purposes such as learning versus blame (Dwyer & Hardy, 2016) or narrative genres like dualism and tragedy (Simko, 2012).

## Conclusion

Our study underscores that people’s actions within a collective response shape their personal experiences of crisis. Our participants did not experience SARS as an abstract threat. Rather, they experienced it through roles within a collective response that could bring their attention to the value of their developing expertise, the moral significance of their actions, or the connections they made in their professional field. By joining in collective action, people can emerge from crisis with stories of growth, guiding and inspiring work that contributes to personal, organizational and societal responses in future crises. Such narratives seem vital as many of the crises which societies face – pandemics, financial crisis and natural disaster among them – are intermittent. Without a narrative thread encouraging professionals to appreciate and cultivate their experiences between crises, the resources they develop in one crisis – including technical and organizational expertise, a sense of purpose, and networks, – may be lost in the years before the next one.

## Appendix

**Table 5.** Second-Order Themes, First-Order Categories and Representative Examples.

Second-order theme	First-order category	Representative examples
Use of expertise *Not implicated*	Assignment based on availability and willingness	I did. Everybody did [*volunteering to screen hospital entrances*]. It didn’t matter who you were. We redeployed large amounts of management staff to what we called ‘managing the door’ [*laughs*] . . . the worst thing was people not coming to work, innocent absenteeism. You know, that was not a problem, people were so committed . . . it’s like ‘every man on board’, everyone’s gotta be manning the ship. (Vera, nursing manager, Downtown – idiosyncratic assignment)
*Utilized and deepened*	Getting experience with something that is emerging	I was in a fortuitous position, since I had the flexibility of a resident to learn and get unique expertise with SARS and I could do research because I didn’t have the regular responsibilities of a staff physician . . . I got a message from [*well-known ID expert*] during a committee meeting that our paper had been accepted. It was one of the highs, if not the highest sense of success in my career, of being able to do this [publishing a paper on SARS] in such an uncertain situation . . . SARS was the foundation of much of my work afterward, for instance in my involvement in pandemic planning I used a lot of my SARS experience. (Anna, medical trainee, Academic – deployment) It's hard to describe the degree of motivation and intensity at a time like that, and the purpose with which people go about their work. And if I try to describe it in florid terms, it would sound dramatic and silly. I’ve just got to tell you, that’s the way it was. (Marshall, physician and medical association manager – deployment) My lesson from SARS was that I had to keep pushing and showing data in order to get buy-in to test [*medical treatment*]. But I thought, ‘If I can do it with SARS, I can do it with Ebola, with Zika, with . . .’ After SARS I did trials of [*treatment*] with Ebola and now we’re working on them for Zika. It’s always a hurdle to push the bias against [*treatment*] but I’ve learned the strategy of amassing data and that demonstrating it will change minds. It’s an evidence-based strategy . . . (Sophia, research scientist, City – deployment)
	Being the only one able to do it	[As an ICU specialist] you’re a bit more practised and a bit more comfortable that you can get in and out of personal protective equipment in a safe way than a lot of practitioners in the hospital that do not have the interaction previously. So an ICU or infectious diseases doctor, if that’s what people do, it forges a greater sense of practice which helps to make you more comfortable that, though there may be some risk involved, you can minimize that risk. (Richard, ICU specialist, Academic – deployment) I was one of the ones that could go in, to help my work. I think it’s your duty to go in as a nurse, to go to the last, to the very end. (Nurse interview [R-CC, p. 804] – deployment)
*Utilized and broadened*	Directing a professional audience	I was seen as a student [at City], because I was a postdoc. [*Her involvement in the SARS committee was initially resisted.*] While at [Suburban], I was seen as someone with expertise and treated [as such] . . . we [the SARS management team] would meet in the war room and communicate. [When I needed something] they would go, ‘whatever you want’. My order sheets would have to get approved by the [Medical Affairs Committee] and they would get it done in a day. So things worked really well. (Anna, medical trainee, City and Suburban – deployment versus steering role) The cancellations [of appointments] gave the psychiatry team a space to create a drop-in centre, which we later replaced with a lounge, so staff could drop in to talk about their issues. We wandered the hallways putting up flyers for our drop-in centre, [reading] ‘we realize you may feel anxious or have insomnia. Feel free to drop by.’ We tried to engage our colleagues in conversation . . . We had some credibility before SARS, which let us do what we did. But our credibility multiplied after SARS . . . [psychiatry] is seen as a routine part of the hospital’s work rather than an unusual or weird case. I think the change is permanent. (William, psychiatrist, Downtown – steering role) [*The psychiatrist’s role expansion quoted above is acknowledged by a senior colleague*]: One of the things that was very helpful to those of us at Downtown Hospital was that our psychiatry department took a huge interest in being available to staff, going out to units to talk to staff, to allow them opportunities to talk about their feelings . . . they didn’t have to do that . . . I think they were very helpful to people, they were very helpful to me. (Vera, nursing manager, Downtown – witnessing a steering role)
Moral position in collective response *Moral inclusion*	Stepping up to do something useful	I don’t want to use the word ‘exciting’, but I was impressed by how staff at all levels, from cleaning staff to front-lines care to the senior physicians and managers pulled together during SARS. (Donald, physician and manager, Academic – witnessing idiosyncratic assignments) What went right: . . . I’d say the people who were going to step up, it was right across the board because it went on and on, they were a smaller group of people involved in stepping up and are then consistently stepping up. But that’s a reflection of professionalism, of human nature. (Physician interview [R-CC, p. 833] – witnessing idiosyncratic assignments)
*Moral centrality*	Bearing the burden	There were only a small number of us working on the SARS wards. And this created a lot of work. An intern [*like myself*] would not normally handle so many patients . . . but I never really questioned it – that’s what physicians do. We look after patients with communicable diseases. And I did feel the higher sense of responsibility I had during SARS. But I had responsibilities I never would have had as an intern and it helped develop me as a physician . . . (Alex, medical trainee, Downtown – deployment) Only those on the inside understood what was happening . . . I was not asked to do this; I was proud to do it. For the foreseeable future, I am a SARS physician. ([MP-PE, p. 282] – deployment) Certainly [SARS] was a formative experience in terms of pride as a unit. When the Ebola thing came around I was at a different point in my career . . . But I think my courage to stand up and say ‘this isn’t going to protect my staff so we’re not going to do it. We’re going to do what’s really protective’, partly that was informed by the SARS experience and realizing later that we were not only asked to do things that only weren’t going to be helpful but were going to be counterproductive. (Henry, ER physician, Downtown – deployment)
*Moral leadership*	Hierarchy flipping over	One guy, quite a high-level guy . . . was asked to see a patient, and he was so scared going in, that Alex [medical trainee, Downtown], as a trainee, had to help him gown up and walk him in. It was a complete role reversal . . . I had huge respect for the ladder, and everything changed after that. (Jennifer, medical trainee, Downtown – witnessing a steering role) The rules were set by some government group, mainly infectious disease doctors. One of them I know, who’s from [another city] said it was quite strange. They advertised that they needed people. They all kind of laughed that ‘Toronto needs us. We couldn’t get a job in Toronto if we wanted to last week, but now they’re flying us here.’ She said ‘They put us in a hotel and every morning we sit in a room and make up rules for you people to work by.’ (Andrew, ICU physician, Downtown – witnessing a steering role)
Basis for post-crisis interactions *Suspended status differences*	Living through a shared ordeal	Relationships with close colleagues . . . there’s a nurse manager of the unit. We became very good friends. We were tasked with something that was incredible . . . [*Gemma and the nurse manager were tasked with purchasing equipment for an improvised SARS unit*] (Gemma, ICU physician, Suburban – idiosyncratic assignment)
	Physical co-presence of medical and non-medical staff	I can tell you that the people who worked in that unit were all extremely dedicated people, that I will work with any time, because it was a risky situation. The staff that cleaned, the housekeeping, did not want to go either . . . The pharmacist was there all the time . . . The infection control nurse . . . was there all day . . . (Physician interview [R-CC, p. 312] – witnessing idiosyncratic assignments)
*Demarcated status differences*	Being on the front lines	There was definitely a sense of solidarity among the people who were engaged in the care of patients, description [of the disease], and research, so there was definitely a relatively focused group of people that were operationally active at the hospital . . . in my interactions at least, it was a system of lateral solidarity among front-line healthcare workers in hospitals that were feeling the burden of engagement of caring for people. (Richard, ICU physician, Academic – deployment) We spent long hours together waiting for the arrival of SARS patients. It was frightening but exciting . . . we spent lots of time sitting around talking to each other, killing time, and finding ways to entertain ourselves . . . I can say the department had a stronger sense of camaraderie that it had before or since . . . we had a shared experience. (Arthur, ER physician, Downtown – deployment)
*Transcended social differences*	Building diverse professional bonds	My qualifications [*for joining a SARS committee*] was that I was the first to accept, and because [*my superior*] was looking for someone who had both a hospital and a community role that could put some common sense into the committee . . . Working through SARS gave me a network with a lot of interesting colleagues and also with government. (Michael, physician and manager, City – steering role) I started doing the [*SARS announcements*] twice daily, seven days a week. And I was never more connected, before or since, to the front-line staff in any organization I’ve worked for. [*In my directives as SARS committee leader*] I tried to be as transparent as possible to reassure staff . . . I worked two more years at City Hospital. [After SARS] it almost didn’t matter if I did anything at City! Because I had already handled SARS . . . SARS was the single most important part of my career learning, especially about communicating. (Stanley, senior manager, City – steering role)
		I got an unexpected opportunity. [*Provincial health authority*] felt it was important that somebody study the clinical features of all SARS patients in the Greater Toronto Area. He said ‘Someone should record this information for the future, for other cities.’ Because the size of the outbreak in Toronto was one of the largest in the world, it was a unique research opportunity . . . he chose me because I had a reputation for getting things done, and because I had already assumed a leadership role at teleconferences [*connecting the SARS committees between Toronto hospitals*]. (David, physician and manager, Downtown – steering role)

## Notes

1. We use pseudonyms for interviewees in this study.

2. We also compared across hospitals and professions in our dataset but did not find this to explain the diverging stories of growth.

3. Many physicians deploying were busy with patients and missed out on career-advancing research projects, one study concluded (MP-STWI, pp. 83–84).

4. Nurses as a group had the most patient contact but we found only about three mentioned by name in the 2,122 pages of the Ontario government’s SARS report, in contrast to SARS committee members (a steering role) who were extensively named (R-CC).
